# Supplementary material for: Fatty acid synthase-derived lipid stores support breast cancer metastasis
Source: Cancer Metab. 2025 Jul 10;13:35. doi: 10.1186/s40170-025-00404-3 (PMC12247306; doi:10.1186/s40170-025-00404-3)
Supplement: Supplementary file 1 — Supplementary Material 1. [file 40170_2025_404_MOESM1_ESM.pdf]

Figure S1

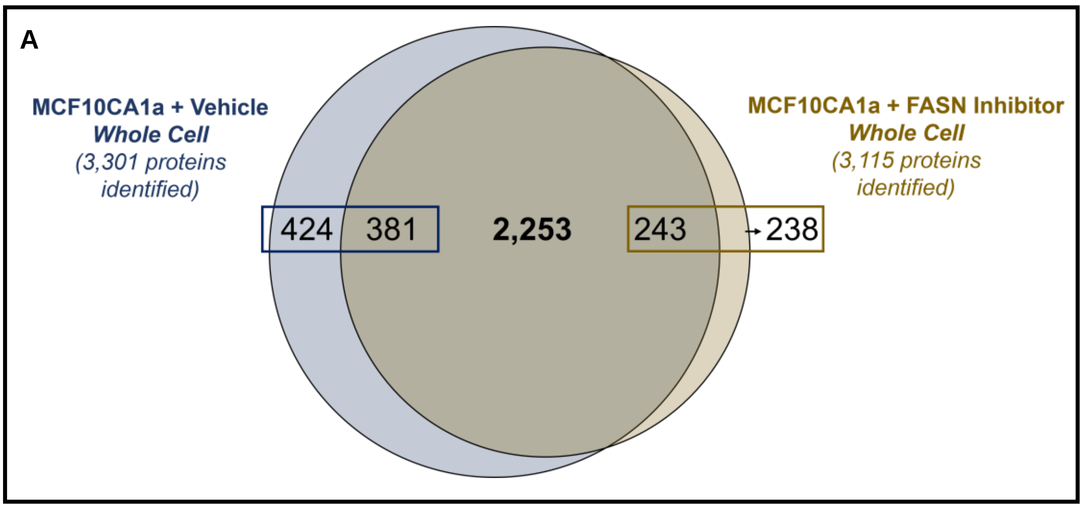

B

	DMSO	TVB-3166
In 2 of 3	536	510
In 3 of 3	2,765	2,605
Total Identified	**3,301**	**3,115**

	WCL
Proteins identified in both	**2,877**
Proteins same LFQ in both	2,253
Proteins different LFQ in both	624
Proteins higher in DMSO	**381**
Proteins higher in TVB-3166	**243**

	WCL
Proteins only in DMSO	**424**
Proteins only in TVB-3166	**238**

	WCL
Total proteins in "DMSO>TVB"	**805**
Total proteins in "TVB>DMSO"	**481**

Fig. S1 Differential abundance of proteins in MCF10CA1a cells treated with vehicle (DMSO) or FASN inhibitor (TVB-3166). **A** Venn diagram depicting proteins identified in the global proteomics analysis that were observed in both conditions (dark tan overlap), proteins found only, or higher, in the vehicle-treated MCF10CA1a cells (boxed in blue), and proteins found only, or higher, in TVB-3166-treated MCF10CA1a cells (boxed in gold). **B** Charts indicating where the number of proteins that comprise A came from. If a protein was identified in 2 of the 3 biological replicates, it was considered present (top panel). Proteins identified in both conditions that are differentially expressed were determined by two-tailed Student's t-test between LFQ value of identified proteins, where $p < 0.05$ (left panel). The sum of differentially expressed proteins (bottom left panel, in color) and proteins identified in only one condition (top right panel) are represented in boxes in both A and B (bottom right panel).

Figure S2

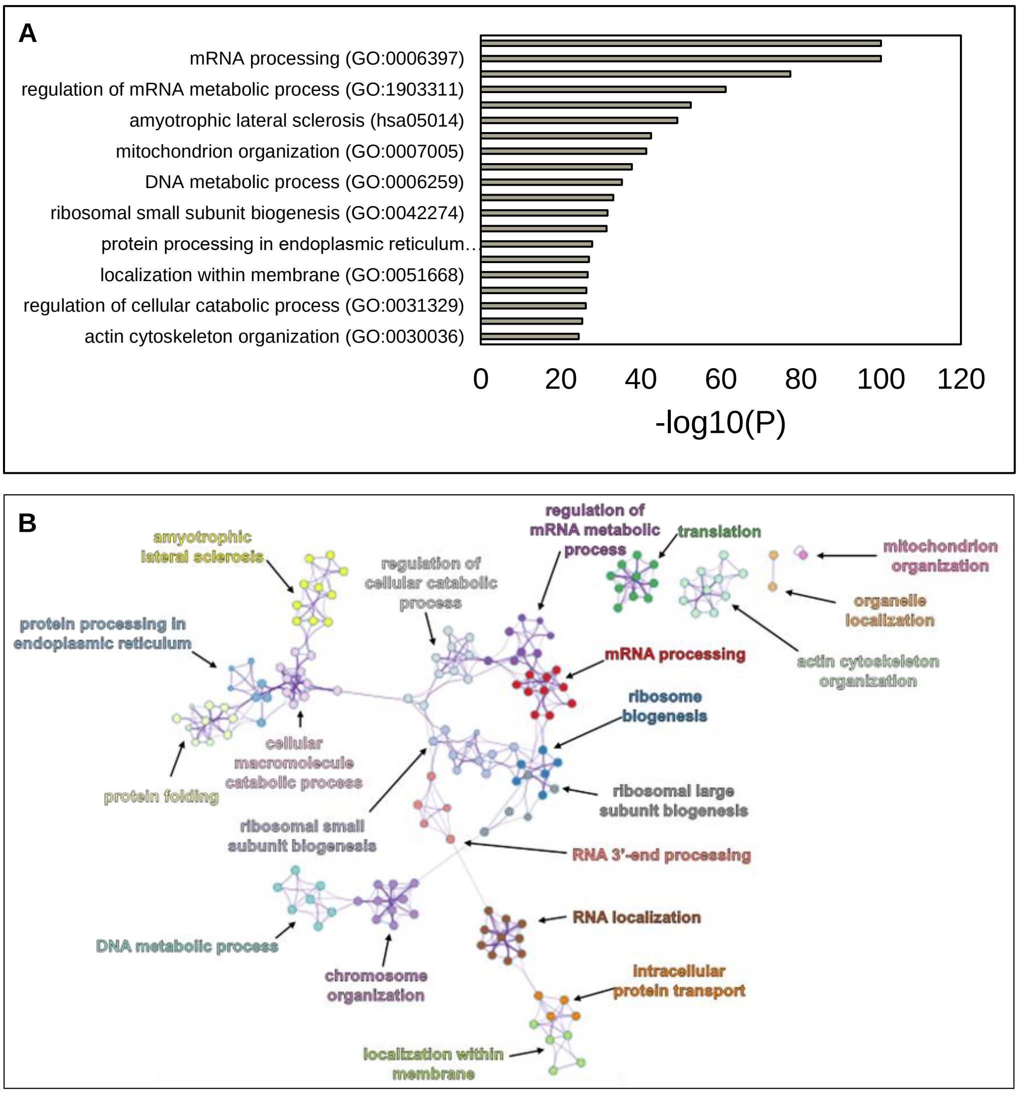

Fig. S2 Functional annotation analysis of proteins present in both FASN-inhibited and vehicle-treated MCF10CA1a cells with similar abundance. **A** Top 20 clusters of statistically enriched Kyoto Encyclopedia of Genes and Genomes (KEGG) pathways and Gene Ontology Biological Process (GO_BP) terms present in FASN-inhibited and vehicle-treated MCF10CA1a cells. The greater the -log10(P) value indicates more enriched terms. **B** Enriched clusters of KEGG pathways and GO_BP terms identified in FASN-inhibited and vehicle-treated MCF10CA1a cells presented in network format. Statistically enriched similar terms are organized into clusters and colored based on the representative parent term for the cluster. Each circle within a colored cluster represents one term, and the size of the circle correlates with the number of proteins identified within that term. Similar terms are connected by a line, with a thicker line indicating higher similarity between terms (calculated in Metascape).

Figure S3

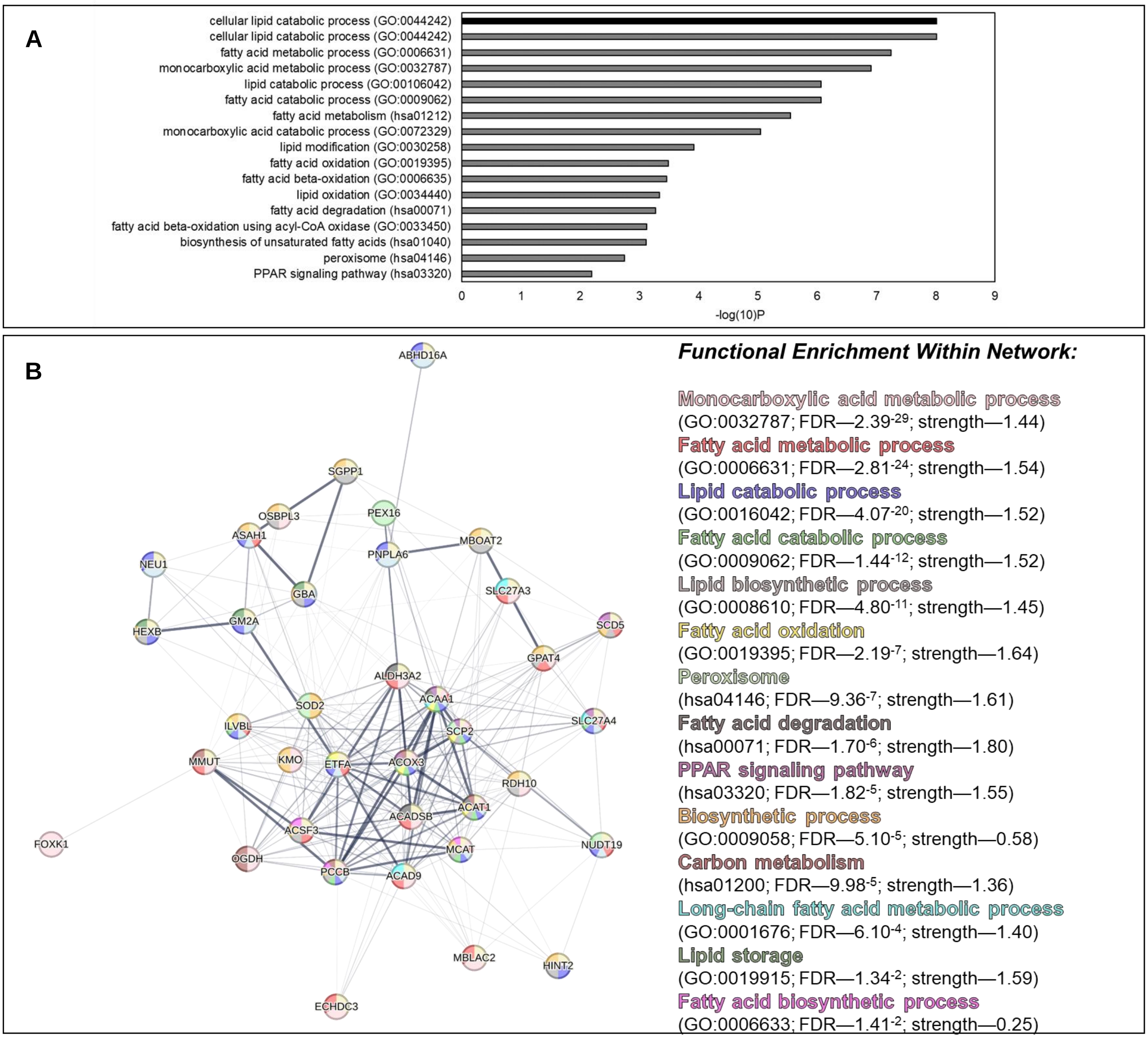

Fig. S3 Annotation of cellular lipid catabolic process proteins that are more enriched in FASN-inhibited than vehicle-treated MCF10CA1a cells. **A** GO_BP and KEGG pathway subcategories “cellular lipid catabolic process”, “fatty acid metabolic process”, “monocarboxylic acid metabolic process”, “lipid catabolic process”, “fatty acid catabolic process”, “fatty acid metabolism”, “monocarboxylic acid catabolic process”, “lipid modification”, “fatty acid oxidation”, “fatty acid beta-oxidation”, “lipid oxidation”, fatty acid degradation”, “fatty acid beta-oxidation using acyl-CoA oxidase”, “biosynthesis of unsaturated fatty acids”, “peroxisome”, and “PPAR signaling pathway” from the parental “cellular lipid catabolic process” GO_BP category (black) from Fig. 5B are visualized in grey. **B** STRING analysis of identified proteins more enriched in FASN-inhibited than vehicle-treated MCF10CA1a cells involved in cellular lipid catabolic processes. Different colors represent functional GO_BP and KEGG enrichments within the searched network. Strength is calculated based on count in searched protein list compared to total present in GO_BP or KEGG network database. All enrichments (the eight different colored categories) have a false discovery rate (FDR) ≤ 0.01. Figure was created and adapted from STRING-db.org (version 12.0). *Abbreviations*: ACAD9 = Complex I assembly factor; ACADSB = Short/branched chain specific acyl-CoA dehydrogenase, mitochondrial; ACAT1 = Acetyl-CoA acetyltransferase, mitochondrial; ACOX3 = Peroxisomal acyl-coenzyme A oxidase 3; ACSF3 = Malonate--CoA ligase ACSF3, mitochondrial; ALDH3A2 = Aldehyde dehydrogenase family 3 member A2; ASAH1 = Acid ceramidase subunit alpha; ECHDC3 = Enoyl-CoA hydratase domain-containing protein 3, mitochondrial; ETFA = Electron transfer flavoprotein subunit alpha, mitochondrial; FOXK1 = Forkhead box protein K1; GBA = Lysosomal acid glucosylceramidase; GM2A = Ganglioside GM2 activator isoform short; GPAT4 = Glycerol-3-phosphate acyltransferase 4; HEXB = Beta-hexosaminidase subunit beta chain A; HINT2 = Histidine triad nucleotide-binding protein 2, mitochondrial; ILVBL = Acetolactate synthase-like protein; KMO = Kynurenine 3-monooxygenase; MBLAC2 = Metallo-beta-lactamase domain containing 2; MBOAT2 = Lysophospholipid acyltransferase 2; MCAT = Malonyl-CoA-acyl carrier protein transacylase, mitochondrial; MMUT = Methylmalonyl-CoA mutase, mitochondrial; NEU1 = Sialidase-1; NUDT19 = Nucleoside diphosphate-linked moiety X motif 19; OGDH = 2-oxoglutarate dehydrogenase, mitochondrial; OSBPL3 = Oxysterol-binding protein-related protein 3; PCCB = Propionyl-CoA carboxylase beta chain, mitochondrial; PEX16 = Peroxisomal membrane protein; RDH10 = Retinol dehydrogenase 10; SCD5 = Stearoyl-CoA desaturase 5; SCP2 = Non-specific lipid-transfer protein; SGPP1 = Sphingosine-1-phosphate phosphatase 1; SLC27A3 = Solute carrier family 27 member 3; SLC27A4 = Long-chain fatty acid transport protein 4; SOD2 = Superoxide dismutase [Mn], mitochondrial.

Figure S4

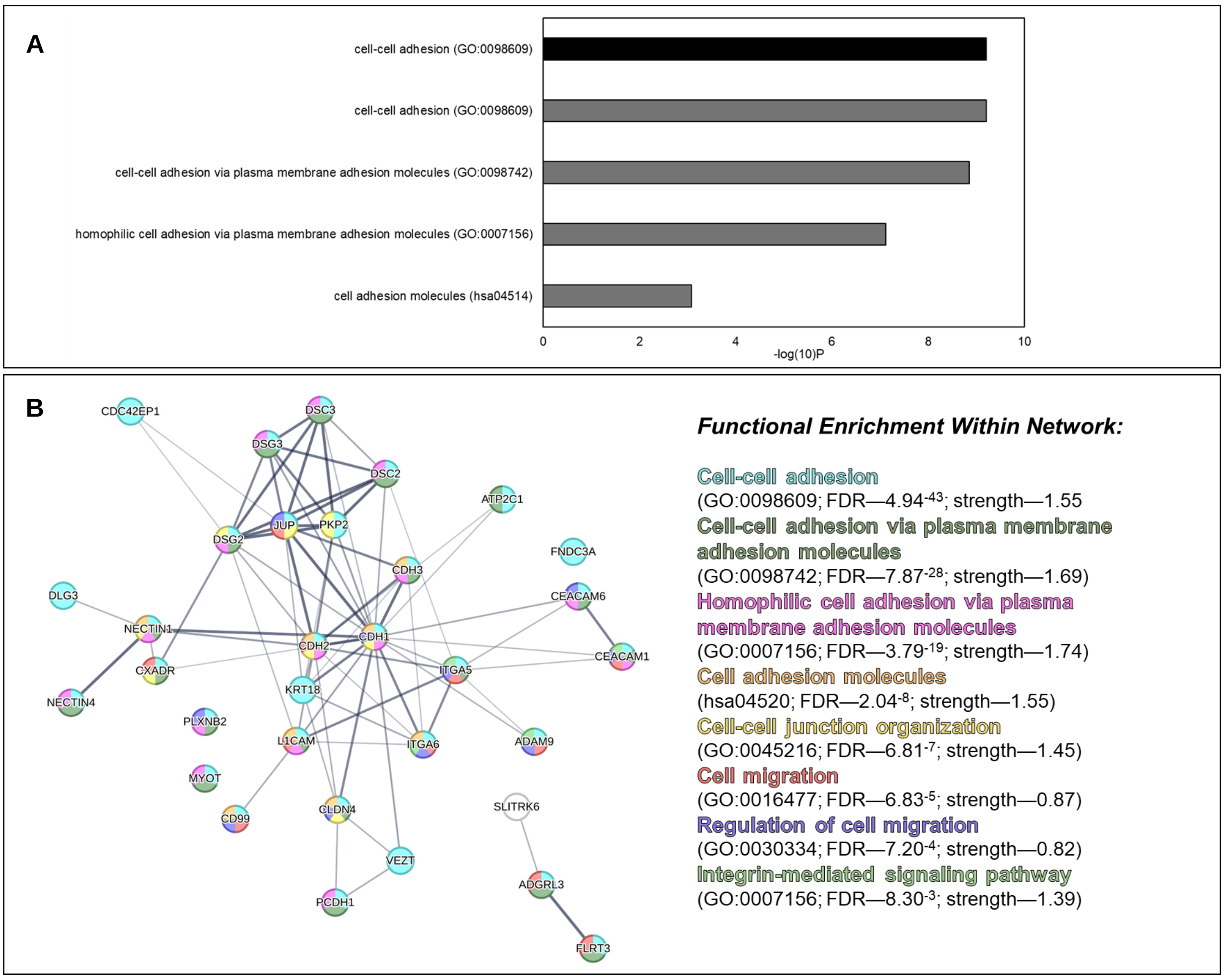

Fig. S4 Annotation of cell-cell adhesion proteins that are more enriched in FASN-inhibited than vehicle-treated MCF10CA1a cells. **A** GO_BP and KEGG pathway subcategories “cell-cell adhesion”, “cell-cell adhesion via plasma membrane adhesion molecules”, “homophilic cell adhesion via plasma membrane adhesion molecules”, and “cell adhesion molecules” from the parental “cell-cell adhesion” GO_BP category (black) from Fig. 5B are visualized in grey. **B** STRING analysis of identified proteins more enriched in FASN-inhibited than vehicle-treated MCF10CA1a cells involved in cell-cell adhesion. Different colors represent functional GO_BP enrichments within the searched network. Strength is calculated based on count in searched protein list compared to total present in GO_BP network database. All enrichments (the eight different colored categories) have a false discovery rate (FDR) ≤ 0.01. Figure was created and adapted from STRING-db.org (version 12.0). *Abbreviations*: ADAM9 = Disintegrin and metalloproteinase domain-containing protein 9; ADGRL3 = Adhesion G protein-coupled receptor L3; ATP2C1 = Calcium-transporting ATPase type 2C member; CD99 = CD99 antigen; CDC42EP1 = Cdc42 effector protein 1; CDH1 = Cadherin-1/E-cadherin; CDH2 = Cadherin-2/N-cadherin; CDH3 = Cadherin-3/P-cadherin; CEACAM1 = Carcinoembryonic antigen-related cell adhesion molecule 1; CEACAM6 = Carcinoembryonic antigen-related cell adhesion molecule 6; CLDN4 = Claudin-4; CXADR = Coxsackievirus and adenovirus receptor; DLG3 = Disks large homolog 3; DSC2 = Desmocollin-2; DSC3 = Desmocollin-3; DSG2 = Desmoglein-2; DSG3 = Desmoglein-3; FLRT3 = Leucine-rich repeat transmembrane protein FLRT3; FNDC3A = Fibronectin type-III domain-containing protein 3A; ITGA5 = Integrin alpha-5; ITGA6 = Integrin alpha-6; JUP = Junction plakoglobin; KRT18 = Keratin, type I cytoskeletal 18; L1CAM = Neural cell adhesion molecule L1; MYOT = Myotilin; NECTIN1 = Nectin-1; NECTIN4 = Nectin-4; PCDH1 = Protocadherin-1; PKP2 = Plakophilin-2; PLXNB2 = Plexin-B2; SLITRK6 = SLIT and NTRK-like protein 6; VEZT = Vezatin
